# Interactions between serum uric acid and gut microbiota: implications for metabolic health

**DOI:** 10.1016/j.metop.2025.100438

**Published:** 2025-12-22

**Authors:** Nurshad Ali

**Affiliations:** Department of Biochemistry and Molecular Biology, Shahjalal University of Science and Technology, Sylhet, 3114, Bangladesh

**Keywords:** SUA, Gut microbiota, Microbiome, Interrelationship

## Abstract

Serum uric acid (SUA), the end product of purine metabolism, is a known risk factor for developing gout; however, recent evidence suggests its broader role in metabolic disorders. The gut microbiota, a complex microbial ecosystem, plays a crucial role in influencing purine metabolism and intestinal uric acid (UA) excretion. Recent findings have uncovered a two-way relationship: certain microbes can metabolize purines and UA, while elevated UA can reduce microbial diversity, alter the production of SCFAs, and compromise intestinal barrier function. These interactions are linked to obesity, insulin resistance, T2D, NAFLD, and CVD, connecting purine metabolism with overall metabolic health. This review synthesizes current experimental and clinical evidence on SUA-microbiota interactions, with an emphasis on microbial enzymes, host urate transporters, and microbial metabolites, including bile acids and SCFAs. It also discusses therapeutic implications, spanning urate-lowering drugs to microbiota-targeted strategies, including probiotics, prebiotics, and dietary modulation. Despite progress, significant gaps remain: most human studies are cross-sectional, microbial taxa influencing SUA remain inconsistent, and interindividual microbiome variability limits the translation of findings to personalized care. Future multi-omics and longitudinal approaches are necessary to elucidate causal pathways and identify biomarkers, ultimately informing innovative strategies for the prevention and treatment of metabolic diseases beyond gout.

## 1 Introduction

Hyperuricemia, defined by high serum uric acid (SUA) levels, is increasingly recognized as a global health concern, with prevalence rates estimated to range from 11 % to 20 % in the general population, and higher figures observed among individuals with metabolic comorbidities [1,2]. While, classically recognized as the primary risk factor for gout, sustained high levels of SUA have also been consistently associated with a wide range of metabolic disorders, such as obesity, insulin resistance (IR), type 2 diabetes (T2D), non-alcoholic fatty liver disease (NAFLD), chronic kidney disease (CKD), and cardiovascular disease (CVD) [3,4] and risk of all cause of mortality [5,6]. Collectively, these conditions represent a significant public health challenge, contributing to increased morbidity, mortality, and healthcare expenses globally. Consequently, it has become important to explore both the upstream factors and downstream effects of uric acid (UA) dysregulation within both basic and clinical research contexts.

In recent years, the gut microbiota—the intricate and dynamic collection of microorganisms residing in the human intestine—has been identified as a crucial factor influencing host metabolism and immune stability [[7], [8], [9]]. Apart from its roles in nutrient absorption and energy regulation, the microbiota plays a part in purine metabolism and UA regulation, particularly through microbial enzymes such as uricase and xanthine dehydrogenase, which are capable of breaking down UA or its precursors [10]. Furthermore, the intestinal tract acts as a significant pathway for UA elimination, supporting renal excretion, as there is evidence indicating that certain microbial species can promote intestinal uricolysis and impact systemic SUA levels [11].

On the other hand, persistent hyperuricemia might also alter the gut microbial ecosystem. Studies utilizing experimental models have shown that elevated SUA can lead to intestinal dysbiosis, decrease the production of short-chain fatty acids (SCFAs), and impair gut barrier integrity, thereby fostering low-grade inflammation and IR [10]. These results advocate for a bidirectional relationship between SUA levels and gut microbiota, establishing a metabolic feedback loop that may affect the onset and progression of metabolic syndrome and its associated complications.

Despite the rapidly growing body of research, there remains a limited comprehensive understanding of how SUA and gut microbiota interact to influence metabolic health. Elucidating these interactions is vital for discovering new biomarkers and therapeutic approaches—ranging from conventional urate-lowering drugs to microbiota-focused interventions such as probiotics, prebiotics, and dietary adjustments. Therefore, this review intends to thoroughly investigate the molecular and physiological mechanisms connecting SUA and gut microbiota, summarize findings from both animal and human studies, and underscore future research directions essential for leveraging this interaction in the prevention and management of metabolic disorders.

## 2 Physiology of UA metabolism

UA is the final product of purine breakdown in humans and higher primates, who lack the liver enzyme uricase that usually converts UA into the more soluble allantoin [12]. Consequently, UA exists in greater concentrations than in most other mammals and is primarily excreted via renal and intestinal routes. The UA pool in the body is derived from two main sources: internal purine metabolism and dietary consumption. Internal purines result from the normal turnover of nucleic acids (DNA, RNA) and the de novo synthesis of purine nucleotides. Dietary purines, found in purine-rich foods like red meat, seafood, and some legumes, are absorbed and enter systemic circulation. Both sources converge into a shared catabolic pathway where nucleotides are progressively broken down to hypoxanthine and xanthine, which are the direct precursors of UA [13].

The liver's production of UA is influenced by xanthine oxidoreductase, which can exist in two interchangeable forms: xanthine dehydrogenase (XDH) and xanthine oxidase (XO). XO facilitates the oxidation of hypoxanthine to xanthine and further to UA, producing reactive oxygen species as by-products [12]. This enzymatic process is the primary focus of pharmacological treatments aimed at lowering urate levels, such as allopurinol and febuxostat.

After being produced, UA is expelled through a tightly regulated equilibrium of renal and intestinal excretion. The kidneys handle about two-thirds of the daily urate clearance. Glomerularly filtered urate experiences intricate tubular processing, which includes both reabsorption and secretion. Important transporters in the proximal tubule consist of the urate-anion exchanger URAT1 (SLC22A12) and glucose transporter GLUT9 (SLC2A9), which facilitate urate reabsorption, alongside ABCG2 and NPT1/4, which aid in secretion [12,13]. Extra-renal elimination takes place through the intestines, where UA is secreted into the gut lumen and decomposed by microbial enzymes such as uricase, highlighting the importance of gut microbiota in maintaining systemic UA balance [14].

Under normal physiological conditions, about one-third of total body UA is excreted through the gastrointestinal tract [15,16]. This process mainly involves urate transporters like ABCG2, SLC2A9 (GLUT9), and SLC22A12 (URAT1) also found in intestinal epithelial cells [15,17]. During metabolic stress or diseases such as obesity, IR, diabetes, and CKD, changes in these transporters and the function of the intestinal barrier significantly impact UA levels [18,19]. Increased oxidative stress and inflammation raise ABCG2 expression in the small intestine as a response to improve UA clearance outside the kidneys [20]. However, conditions like metabolic endotoxemia, intestinal dysbiosis, and damage to the epithelium can impair this pathway, causing urate to build up in the system [21]. At the same time, higher fructose metabolism and XO activity in enterocytes increase local production of UA, putting more pressure on the transport system [22]. In CKD and metabolic syndrome, the intestines handle urate excretion becomes an important fallback when kidney clearance decreases, but ongoing inflammation may reduce its effectiveness [23]. Therefore, the intestines play a vital role in managing SUA levels in the body, especially in relation to various diseases.

Any disruption of this system—whether through excessive purine intake, heightened XO activity, diminished renal or intestinal clearance, or dysbiosis affecting microbial uricolysis—can lead to elevated SUA levels and trigger hyperuricemia. Such increases not only make individuals susceptible to gout but are also increasingly recognized as factors in IR, CVD, and other metabolic conditions. Therefore, comprehending the integrated physiology of UA metabolism, especially the dual roles played by renal transporters and gut microbial breakdown, is essential for understanding the development of metabolic diseases linked to hyperuricemia.

## 3 Gut microbiota and purine/UA metabolism

The human gut contains a rich and varied microbial community primarily consisting of bacteria from the phyla Firmicutes, Bacteroidetes, Actinobacteria, and Proteobacteria, along with archaea, fungi, and viruses (Table 1) [8]. This microbiome is essential for digestion, energy extraction, immune regulation, and the transformation of various dietary and host-derived compounds [8]. Among its metabolic roles, the gut microbiota has been identified as a significant player in purine metabolism and the control of systemic UA levels.

**Table 1:** Key gut microbial taxa and species-level evidence in purine/uric-acid metabolism.

Microbial taxa (genus/species)	Metabolic function (uricase/purine degradation)	Effect on SUA levels	Supporting evidence (animal/human) — species-level	Reference
Lactiplantibacillus plantarum (specific strains studied)	Ribonucleoside hydrolases (RihA–C) hydrolyze nucleosides → reduce precursor absorption and hepatic urate synthesis	Decrease (reduced serum urate in mice)	High-nucleoside diet mouse models; genomic & recombinant enzyme assays demonstrating nucleoside degradation and reduced hepatic XOD activity	[35]
Lacticaseibacillus paracasei JS-3	Direct UA degradation and purine metabolism modulation (in vitro UA degradation; increases SCFAs)	Decrease (lowered serum urate; increased fecal UA degradation)	Isolated from fermented food; hyperuricemic quail model showing normalization of SUA and renal protection after JS-3 administration	[30]
Lacticaseibacillus (Lactobacillus) rhamnosus GG (LGG)	Genome-encoded nucleoside/purine-degrading genes (e.g., iunH, pbuX); reduces intestinal nucleoside absorption and enhances gut-liver-kidney axis urate excretion	Decrease (ameliorates diet-borne hyperuricemia in animal model)	Novel diet-induced hyperuricemia goose model; LGG intervention improved SUA, reduced hepatic/renal inflammation and altered purine-metabolism genes	[71]
*Bifidobacterium longum* (and other Bifidobacteria species)	Expression of purine-degrading enzymes; modulation of intestinal inflammation and urate excretion pathways (e.g., ABCG2 regulation)	Decrease (probiotic strains associated with lower SUA and improved renal/immune markers in animal studies)	Multiple rodent models and probiotic intervention studies showing Bifidobacterium enrichment reduces hyperuricemia-related inflammation and improves urate handling	[64]
*Escherichia coli* Nissle 1917 (wild-type & engineered strains)	Native anaerobic UA metabolic pathways; engineered strains expressing urate oxidase/importer (PucL/PucM, ygfU) and oxygen-recycling modules for enhanced UA degradation	Decrease (engineered and wild-type EcN degrade UA in vitro and lower SUA in hyperuricemic mice)	In vitro anaerobic UA degradation assays and engineered EcN oral administration in mouse hyperuricemia models showing significant UA lowering	[69]
Diverse anaerobic gut bacteria carrying urate/purine-degrading gene clusters (community-level taxa)	Bacterial gene clusters for urate degradation, nucleoside hydrolases and purine catabolism; community consumption of urate or conversion to other metabolites (e.g., xanthine, SCFAs)	Decrease (community-level consumption of purines/urate associated with lower host SUA in gnotobiotic and metagenomic studies)	Metagenomics, metatranscriptomics, stable-isotope tracing and gnotobiotic mouse colonization experiments identifying urate-degrading gene clusters and functional impact on host urate levels	[42]

**Fig. 1:**
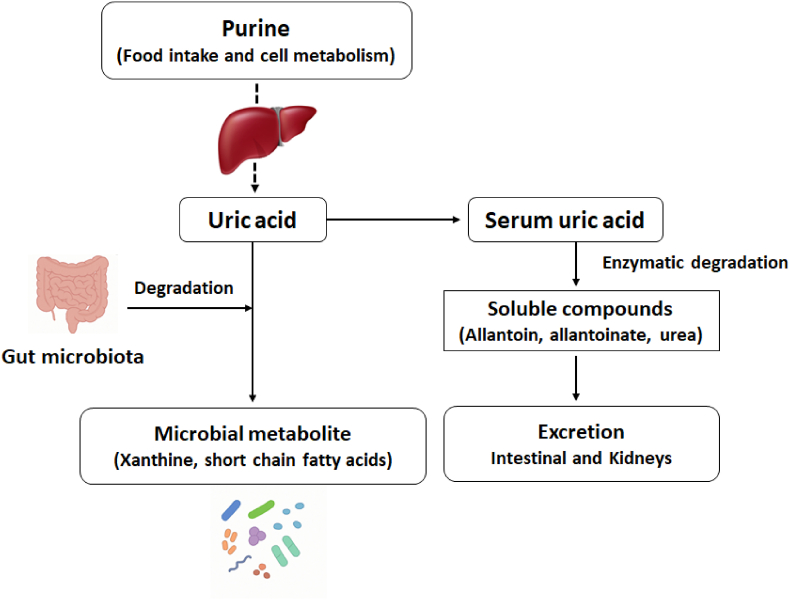
Schematic of uric acid metabolism and gut microbial degradation pathways.

Dietary and endogenous purines that are not absorbed in the small intestine make their way to the colon, where they are further broken down by gut microorganisms (Fig. 1). Certain bacterial groups have enzymes necessary for purine degradation, including xanthine dehydrogenase and, notably, uricase (urate oxidase), which facilitates the conversion of UA into the more soluble compounds such as allantoin, allantoinate, urea, and reactive ROS, which have various metabolic effects [10,24]. In humans, increased ROS from UA oxidation through xanthine oxidase leads to oxidative stress, mitochondrial dysfunction, and damage to blood vessels [25]. This contributes to IR and metabolic inflammation [26]. On the other hand, allantoin—a product of oxidation—has shown antioxidant and anti-inflammatory properties [18]. It may help counteract UA-induced oxidative damage and influence glucose and lipid metabolism [18]. The balance between pro-oxidant and protective metabolites may affect the overall metabolic outcome of UA breakdown. These processes connect UA metabolism with energy balance, blood vessel function, and inflammation, highlighting its role in metabolic disease instead of being just a passive marker.

The gut microbiota influence circulating SUA levels through several mechanisms involved in UA breakdown. First, certain intestinal bacteria produce enzymes that break down UA and its purine precursors. For instance, microbes like *Lactobacillus plantarum* contain ribonucleoside hydrolases that convert nucleosides into nucleobases [27,28]. This process reduces the substrate load for liver UA production, leading to lower SUA levels. Second, the microbial production of short-chain fatty acids (SCFAs), such as butyrate, enhances intestinal barrier integrity and affects host transporter systems [27,29]. Third, gut microbiota can inhibit both the liver and kidneys from producing and reabsorbing UA. For example, probiotic strains have been shown to lower hepatic xanthine oxidase activity and reduce the activity of renal urate reabsorption transporters like URAT1 and GLUT9, which contributes to decreased SUA levels [27,30]. Finally, an imbalance in gut microbiota can impair these protective mechanisms and reduces the ability to break down UA, disrupts intestinal barrier function, increases inflammation, and lowers transporter-mediated excretion leading to higher SUA levels [27,28].

## 4 Impact of UA on gut microbiota

Effects of UA on gut microbiota: An increasing amount of experimental and clinical research suggests that high SUA levels and hyperuricemia have concrete impacts on the diversity, composition, and functionality of gut microbiota. These alterations in microbiota subsequently influence host metabolic and inflammatory pathways, establishing a cycle that may encourage metabolic dysfunction.

### 4.1 Influences on diversity and dysbiosis

Multiple studies indicate that hyperuricemia correlates with diminished microbial diversity and shifts in composition indicative of dysbiosis. In both animal models and human populations, researchers frequently find a decrease in beneficial, SCFA–producing taxa (such as members of Faecalibacterium, Roseburia, and certain Lachnospiraceae) along with a relative increase in pathobionts, including numerous Proteobacteria and opportunistic species from Enterobacteriaceae [8,31]. A decline in alpha diversity and a reduction in SCFA producers are significant because SCFAs (like butyrate and propionate) are vital for maintaining epithelial health and regulating host energy and immune balance; their decrease is often associated with metabolic inflammation and IR [8,32].

### 4.2 Experimental and clinical evidence for taxonomic changes

Animal studies (Table 2): In rodent hyperuricemia models, either induced pharmacologically or through excessive dietary purine, researchers have consistently observed changes in microbiota: a reduction in the abundance of obligate anaerobic SCFA producers and an increase in facultative anaerobes [31,33]. Some experimental studies in animals indicate that lowering urate levels (for example, via xanthine oxidase inhibition or introducing uricase-producing bacterial strains) can partially restore a healthier microbiome profile and improve metabolic phenotypes, implying a causal relationship [27,30,34,35].

**Table 2:** Evidence from animal and human studies on SUA–microbiota interactions.

Study type	Intervention/Condition	Major findings (microbial shifts, SUA changes)	Impact on metabolic outcomes	Reference
Animal (mouse/quail)	Oral administration of Lacticaseibacillus paracasei JS-3 (probiotic) in diet-induced hyperuricemia	Restored gut-community composition (↑ beneficial Lactobacilli, Bifidobacteria), increased fecal uric-acid degradation, reduced SUA.	Improved kidney markers, reduced oxidative stress and inflammation; amelioration of hyperuricemia phenotype.	[30]
Animal (mouse)	Engineered *Escherichia coli* Nissle 1917 expressing urate-degrading genes (PucL/PucM) delivered orally	Engineered EcN colonized gut and significantly lowered SUA vs controls; evidence of in-gut urate degradation.	Lowered hyperuricemia and related kidney injury; proof-of-concept for microbiome-based urate therapy.	[69]
Animal (mouse/rodent models)	Probiotic strains (various Lactobacillus/Bifidobacterium) or compound probiotics in hyperuricemic models	Enrichment of SCFA-producing taxa, reduced xanthine oxidase activity, decreased SUA and fecal microbiota shifts toward “healthy” profile.	Attenuation of hepatic steatosis, lower systemic inflammation, improved insulin sensitivity proxies.	[64]
Animal (mouse)	High-fructose feeding (dietary model of hyperuricemia) altering microbiota	Fructose increased SUA and promoted dysbiosis (↑ pathobionts, ↓ SCFA producers); SUA amplified fructokinase expression and lipogenesis.	Development of hepatic steatosis, IR and metabolic syndrome features.	[22]
Clinical — observational (human cohorts/metagenomics)	Cross-sectional/prospective analyses of gut microbiota in hyperuricemia/gout vs controls	Distinct microbiota signatures in HUA/gout (reduced uricolytic taxa, altered purine metabolism gene abundance); baseline microbiome features predicted HUA in some cohorts.	Associations with metabolic syndrome components; microbiome signatures correlated with SUA and gout risk.	[52]
Clinical — interventional (human trials/meta-analysis)	Probiotic supplementation trials and pooled analyses (various Lactobacillus/Bifidobacterium combinations)	Several small trials and a recent meta-analysis reported modest but significant reductions in SUA and improvements in liver/renal markers vs placebo.	Improvements in metabolic biomarkers (ALT, inflammation); heterogenous trial designs and short durations—evidence still emerging.	[63].
Animal → translational (multi-omics/gnotobiotic)	Metagenomics + stable-isotope tracing + gnotobiotic transfers to test microbiota capacity to metabolize purines/urate	Identification of bacterial gene clusters for purine/urate degradation; colonization with specific communities reduced host SUA in germ-free mice.	Demonstrates community-level capacity to consume host urate and modify metabolic phenotype (proof for causal microbiota → SUA effects).	[42]

Human studies: Data from human studies are more varied but generally support these findings (Table 2). Cross-sectional studies of individuals suffering from hyperuricemia or gout reveal modifications in community structure compared to normouricemic controls—showing reduced microbial richness and shifts in various genera involved in carbohydrate fermentation and purine metabolism [33,36]. Genetic investigations pointing to ABCG2 and other urate-processing genes suggest that diminished intestinal urate secretion may correlate with specific microbiome profiles, although longitudinal and interventional data on humans remain scarce [37,38].

### 4.3 Mechanistic insights: inflammation and barrier integrity

Mechanistic studies indicate several interconnected pathways through which increased UA disrupts the gut ecosystem and its interaction with the host: Pro-inflammatory signaling. UA (especially in its crystalline form) acts as a strong activator of the NLRP3 inflammasome, stimulating the release of interleukin-1β and other cytokines from immune cells [39,40]. Even soluble urate can induce oxidative stress and a pro-inflammatory response in epithelial and immune cells. This mucosal inflammation may foster an environment conducive to the proliferation of inflammation-resistant taxa (e.g., Proteobacteria) while inhibiting obligate anaerobes, resulting in a shift towards dysbiosis [3,39].

#### 4.3.1 Intestinal barrier dysfunction

Hyperuricemia and the inflammation caused by urate are connected to compromised tight-junction integrity, leading to increased intestinal permeability in animal models. This barrier breakdown allows microbial products like lipopolysaccharide (LPS) to enter the bloodstream (metabolic endotoxemia), exacerbating systemic inflammation and IR—a crucial mechanism linking gut dysbiosis to metabolic disorders [8,32].

#### 4.3.2 altered microbial metabolism

Changes in community composition lead to functional shifts: a decrease in microbial uricolysis (loss of uricase functionality in the gut), reduced SCFA production, and modified bile acid metabolism. These functional changes impact host energy absorption, signaling through G-protein coupled SCFA receptors, and enterohepatic bile acid pathways—each of which may affect glucose and lipid metabolism [8,32].

Collectively, the available preclinical and observational studies on humans indicate that hyperuricemia leads to disturbances in gut microbiota and impaired barrier function, which in turn trigger low-grade inflammation and metabolic issues. Nevertheless, the majority of human research conducted so far is cross-sectional, making it impossible to determine cause and effect. There is a significant lack of interventional human trials aimed at lowering urate levels or modifying the microbiome, especially those with coordinated microbiome and metabolomic outcomes, which are essential for establishing causal relationships and pinpointing potential therapeutic targets.

## 5 Bidirectional interactions and mechanistic pathways

**Fig. 2:**
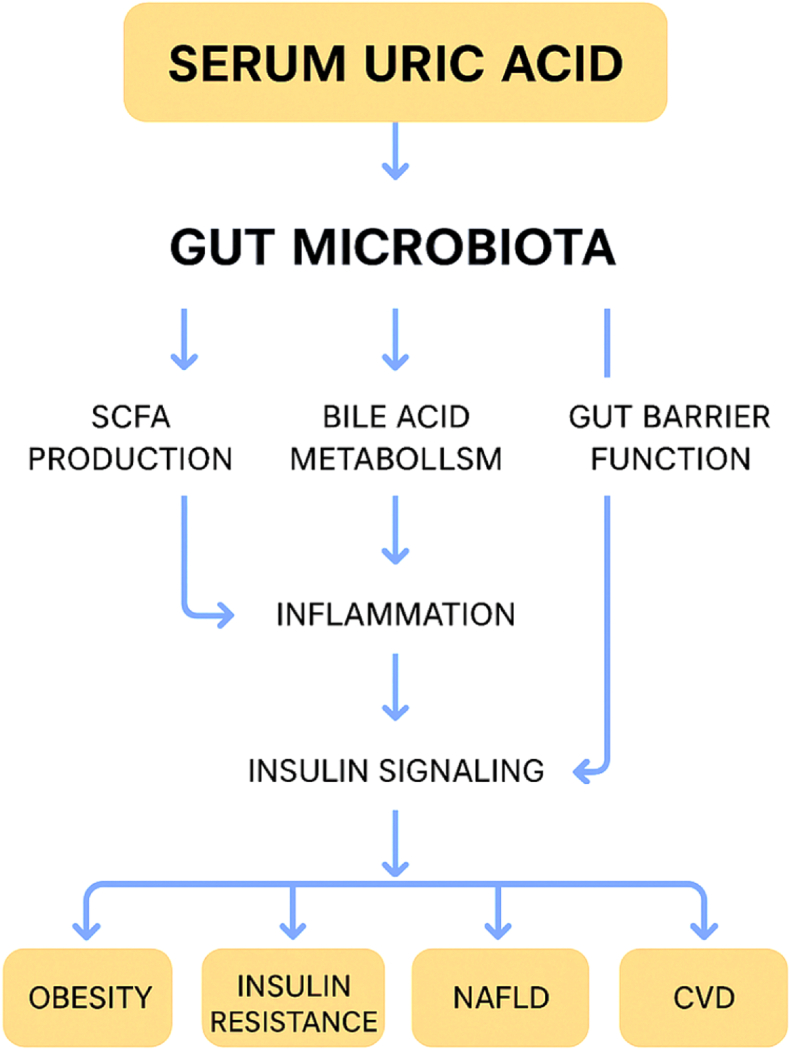
Mechanistic pathways linking SUA–microbiota axis to metabolic disorders.

The relationship between SUA and gut microbiota is reciprocal (Fig. 2), influenced by various co-metabolic, signaling, and transport mechanisms that affect the metabolic health of the host. Here, we outline essential mechanistic themes: microbe-host purine/SCFA co-metabolism, signaling through bile acids and other metabolites derived from the gut (including immune modulation), and the functions of intestinal transporters and enterohepatic pathways.

### 5.1 Microbe–host co-metabolism of purines and SCFAs

Gut bacteria are equipped with enzymatic processes for breaking down purines and, in certain instances, can either directly consume UA or convert it into other products (such as xanthine, allantoin, or SCFAs). Research utilizing stable-isotope tracing and multi-omics approaches has demonstrated that bacterial gene clusters, widely found among anaerobic species, can partially compensate for humans' lack of uricase by converting urate into xanthine or SCFAs, thus reducing the host's urate levels [35,41,42]. Research also highlighted that removing gut microbes in uricase-deficient mice causes severe hyperuricemia, and using antibiotics that kill anaerobic bacteria increases the risk of gout in humans [41]. Experimental studies using colonization and genotobiotic transfers have validated that specific microbial communities can decrease circulating SUA in germ-free mice, indicating a causal role of microbes in purine uptake.

Recent evidence suggests intestinal bacteria such as such as *Lactobacillus reuteri*, *Lactobacillus fermentum*, *Clostridium* spp., and *Escherichia coli-*produce uricolytic enzymes that degrade uric acid, or its precursors, into soluble and less toxic compounds which can be more easily excreted through the gut rather than the kidneys [11,14]. As a result, enhanced uric acid degradation in the gut may lower the amount of uric acid reabsorbed into the bloodstream. This can decrease SUA levels and reduce the risk of hyperuricemia. This microbial “extrarenal” pathway represents offers a potential target for therapeutic intervention [27].

### 5.2 Role of bile acids, gut-derived metabolites, and immune modulation

The gut microbiota alters the bile-acid composition through processes like deconjugation and dehydroxylation, leading to the production of secondary bile acids that interact with FXR, TGR5, and other receptors to modulate lipid and glucose metabolism in the liver as well as systemic inflammation. Consequently, changes in bile-acid signaling create a mechanism through which dysbiosis can affect hepatic purine metabolism and increase the risk of metabolic disorders. [43,44]. At the same time, microbial by-products like hippurate and other co-metabolites can impact host immunity and metabolic characteristics; activation of the inflammasome and low-grade inflammation in the intestines (which may be triggered by dysbiosis or urate crystals) can further disrupt insulin signaling and modify natural handling of metabolites in the enterohepatic system [36,45].

### 5.3 Transporters and enterohepatic circulation

Urate transporters within the intestine and kidneys (including ABCG2, SLC2A9/GLUT9, URAT1, and members of the OAT family) create a widespread network that controls urate movement between the bloodstream, bile/intestine, and urine. The intestine plays a significant role in urate excretion through mechanisms such as ABCG2 and other transporters, with microbial metabolites (like SCFAs) and host signaling pathways influencing the expression and activity of these transporters [46,47]. Emerging research suggests that interventions aimed at the microbiota (such as probiotics and dietary changes) can impact the expression of transporters like ABCG2/GLUT9 and OATs, thereby altering the handling of urate in the intestines and affecting systemic SUA levels [23,27].

A recent study identifies *Alistipes indistinctus* as an important gut microbe that is reduced in people with hyperuricemia [48]. Through combined metagenomic and metabolomic analyses, the authors demonstrate that *A. indistinctus* lowers uric acid levels by producing hippuric acid. Hippuric acid improves PPARγ binding to the ABCG2 promoter, which increases the excretion of urate in the intestines. It also helps ABCG2 move to the brush border membranes via PDZK1. These findings reveal a microbial pathway where *A. indistinctus* and its metabolite hippuric acid regulate uric acid balance.

## 6 Implications for metabolic health

The bidirectional connection between SUA and the gut microbiota has significant implications for metabolic health, especially regarding dyslipidemia, metabolic syndrome, IR, T2D, gout, and CVD [18,49,50]. Hyperuricemia has been repeatedly linked to the components of metabolic syndrome, including central obesity, dyslipidemia, hypertension, and impaired glucose tolerance, which together elevate the risk of CVD and T2D [18,40,49]. In patients with hyperuricemia and gout, dysbiosis is marked by a decrease in uricolytic taxa and a reduction in SCFA-producing bacteria, leading to increased systemic inflammation and metabolic disturbances [31,51,52].

Emerging evidence suggests that elevated SUA play a role in the development of IR (IR) through several pathways. First, elevated SUA increases oxidative stress in endothelial and fat tissues, which lowers nitric oxide (NO) availability and hinders insulin-stimulated glucose uptake [53]. Second, SUA activates pro-inflammatory signals like NF-κB and the NLRP3 inflammasome. This activation leads to increased cytokine release, such as TNF-α and IL-1β, and causes fat cell dysfunction, driving IR [54,55]. Third, SUA might disrupt insulin signaling by preventing IRS-1 phosphorylation and downstream Akt activation. This disruption weakens insulin's effect on metabolism in muscle and liver [26]. Additionally, SUA is associated with the buildup of visceral fat, which in turn worsens IR [26]. While there are bidirectional relationships (IR also decreases urate excretion and increases SUA), mechanistic studies support that SUA causes IR. Reducing SUA has been shown to improve insulin sensitivity in some trials, indicating potential for treatment.

Understanding the interactions is crucial through the lens of the gut–liver–kidney axis. The intestine plays a key role in the excretion of urate, facilitated by transporters such as ABCG2 and SLC2A9. Changes in microbial metabolites can affect these transporters and liver purine metabolism, thereby impacting SUA homeostasis directly [46,47]. On the other hand, hyperuricemia leads to oxidative stress, endothelial dysfunction, and the activation of the inflammasome, which subsequently disrupt insulin signaling and lead to hepatic steatosis and kidney damage [22,[56], [57], [58]].

As discussed above, T2D and IR, increased SUA worsens the impairment of glucose uptake by inhibiting the PI3K/AKT pathway, while changes in the microbiome also add to lipotoxicity and alter bile acid signaling [59,60]. In CVD, the vascular dysfunction induced by SUA is aggravated by systemic inflammation driven by dysbiosis, raising the risk of atherogenic events [18,61]. Gout serves as the most immediate clinical evidence of SUA–microbiota interplay, where the loss of microbial uricase and the consumption of dietary purines come together to enhance the deposition of monosodium urate crystals [18,43].

## 7 Therapeutic and preventive strategies

Targeting the relationship between SUA and the microbiota presents new possibilities for preventing and managing metabolic conditions. Dietary modifications are still the primary strategy. Diets low in purines and reduced fructose consumption lower UA production, while high-fiber diets promote microbial fermentation and the production of SCFAs, enhancing intestinal barrier function and the excretion of urate [62]. Probiotics like Lactobacillus and Bifidobacterium have demonstrated urate-lowering properties in animal studies and initial human trials by increasing uricolytic and SCFA-producing bacteria, whereas prebiotics such as inulin support the growth of beneficial microorganisms [63,64].

Clinical UA-lowering agents, such as xanthine oxidase inhibitors (like allopurinol and febuxostat) and uricosuric drugs (including benzbromarone and lesinurad), provide metabolic benefits beyond just lowering urate [18,65]. By blocking xanthine oxidase, these agents reduce the production of reactive ROS, improve endothelial function, and increase insulin sensitivity [18,65]. Allopurinol has been shown to lower fasting insulin levels and improve glucose uptake in people with metabolic syndrome [18,65,66]. Febuxostat may also enhance lipid profiles and reduce systemic inflammation by lowering CRP and TNF-α levels [67]. Uricosurics boost UA excretion through the kidneys, which can indirectly improve kidney and heart health [18]. However, the extent of metabolic improvement varies among different patients and depends on how long they are treated. Overall, these medications are the standard therapies for managing hyperuricemia and gout. However, new findings suggest that ULT might also alter gut microbiota composition. For instance, febuxostat decreased intestinal urate accumulation and changed gut microbial diversity in hyperuricemic mice, indicating possible interactions between drugs and the microbiota [68]. While these medications effectively reduce SUA, their long-term impact on microbiota-related metabolic regulation is still inadequately studied.

Strategies that target the microbiota represent a growing area of therapeutic innovation. Fecal microbiota transplantation (FMT) has been experimentally investigated to restore uricolytic ability and decrease hyperuricemia, although clinical evidence remains limited [10,64]. Advanced probiotics, such as genetically modified *Escherichia coli* Nissle 1917 that produce urate-degrading enzymes, have shown promising outcomes in animal models, resulting in lowered SUA and improved kidney damage [69]. These engineered strains illustrate a precision microbiome approach with potential for real-world application.

In summary, combining dietary management, traditional urate-lowering medications, and microbiome-targeted therapies could create a synergistic approach to tackle hyperuricemia and its associated metabolic issues. Future investigations should concentrate on controlled clinical studies to assess the effectiveness, safety, and sustainability of these integrated methodologies.

## 8 Current challenges and knowledge gaps

Despite increasing evidence the relationship between SUA and gut microbiota to metabolic health, several significant challenges obstruct the translation of these findings into clinical practice.

Limitations of current research: Evidence from human studies is increasing but varies widely: observational metagenomic research consistently identifies unique microbiome signatures in hyperuricemia/gout, whereas clinical trials involving probiotics show slight reductions in SUA but have differing levels of study quality and duration [52]. More extensive randomized controlled trials are necessary. Moreover, the majority of the existing evidence comes from cross-sectional human studies or brief animal experiments. While these studies highlight important correlations, they fall short of establishing causal relationships. For example, the differences in microbiota noted in cohorts with hyperuricemia and gout might reflect outcomes rather than the causes of altered SUA metabolism [52,70]. Although animal studies provide valuable mechanistic insights, they typically use high-dose dietary or pharmacological models that do not accurately mimic human hyperuricemia and its associated conditions [22,62]. Additionally, significant variability in microbial composition among individuals complicates the ability to generalize findings across different populations.

Need for longitudinal, mechanistic, and interventional studies: There is a scarcity of long-term prospective cohort studies that integrate SUA, microbiome changes, and metabolic results. Only a limited number of interventional trials have systematically evaluated whether microbiome-targeted approaches, such as probiotics, prebiotics, or engineered microbes, can effectively lower SUA levels and enhance metabolic outcomes in humans [63,69]. Furthermore, there is a lack of mechanistic studies investigating how microbial metabolites—such as SCFAs, bile acids, and products from purine degradation—affect host urate transporters and immune responses. Multi-omics strategies that merge genomics, transcriptomics, metabolomics, and stable-isotope tracing could help clarify these causal relationships.

Standardization and methodological integration: A significant obstacle to progress is the absence of standardized methods for microbiome analysis. Differences in sample collection techniques, sequencing technologies, and bioinformatics methodologies impede reproducibility and the ability to compare results across studies. Additionally, few investigations link microbiome data with metabolomics and host transcriptomics to capture the intricate nature of SUA–microbiota interactions. There is an urgent need for standardized frameworks that correlate microbial taxa with functional metabolic pathways to identify clinically relevant biomarkers and therapeutic targets.

In conclusion, while interactions between SUA and microbiota are becoming recognized as key factors influencing metabolic health, the existing evidence is still disjointed. To bridge these knowledge gaps, it is essential to conduct well-structured longitudinal cohort studies, mechanistic studies that span from animal models to humans, and standardized, integrative multi-omics approaches. Such initiatives are crucial for progressing from mere associations to precise interventions that utilize the SUA–microbiota relationship for the prevention and treatment of metabolic diseases.

## 9 Future directions

Future studies examining the SUA–microbiota connection should focus on integrative methodologies that shift from mere associations to a deeper understanding of mechanisms and translational applications. Utilizing multi-omics techniques—including metagenomics, metabolomics, and host transcriptomics—offers significant potential. By characterizing microbial species, functional gene pathways, and circulating metabolites concurrently, multi-omics can elucidate how specific microbial populations influence purine metabolism, short-chain fatty acid (SCFA) production, and bile acid signaling, thereby affecting urate transport and systemic inflammation. The combination of stable-isotope tracing with omics techniques may also help clarify causal pathways in host–microbe purine co-metabolism.

These methodologies pave the way for personalized medicine advancements. Considering the considerable variability among individuals in their gut microbiota composition and urate-handling genetics (such as polymorphisms in ABCG2 and SLC2A9), customizing interventions based on microbiome and genetic profiles could improve treatment outcomes. For instance, engineered probiotics that produce uricolytic enzymes or targeted dietary approaches may be tailored for patients with particular microbiome characteristics or transporter gene variants.

A further area of exploration is the discovery of biomarkers for early detection and risk assessment. Microbial gene patterns linked to purine breakdown, metabolites in feces or plasma like SCFAs and secondary bile acids, along with combined host–microbiota metabolite profiles, may act as predictive indicators for conditions related to hyperuricemia, such as gout, IR, and CVDs. Incorporating these biomarkers into clinical settings could facilitate earlier interventions and more accurate risk management.

In summary, progressing from mere descriptive correlations to predictive, personalized frameworks necessitates longitudinal studies, mechanistic research, and thorough validation of biomarkers across varied populations.

## 10 Conclusions

The relationship between SUA and the gut microbiota is complex and has a significant influence on metabolic health. The microbiota contributes to the metabolism of purines, the breakdown of urate, and the production of regulatory metabolites such as SCFAs and bile acids. In turn, elevated SUA levels can disrupt microbial communities, promote dysbiosis, and trigger systemic inflammation. This interactive relationship links hyperuricemia to various metabolic disorders, including gout, IR, T2D, and CVDs, highlighting the gut-liver-kidney axis as a crucial center for metabolic regulation. From a clinical standpoint, understanding this axis opens up new opportunities for prevention and treatment. It is essential to recognize that traditional urate-lowering medications may inadvertently impact microbial composition. Additionally, dietary modifications, probiotics, and advanced engineered probiotics present promising strategies to address both SUA levels and the microbiota simultaneously. However, significant knowledge gaps remain. Most existing human data are cross-sectional, the underlying mechanisms are not well understood, and methodological differences complicate reproducibility. Future research should focus on longitudinal and interventional studies that incorporate multi-omics strategies and validate microbiome-based biomarkers for risk assessment and management. Ultimately, advancements in this field could enhance precision medicine approaches that not only lower SUA levels but also restore microbial balance, helping to reduce the prevalence of metabolic disorders.

## Conflict of interest

The author has no conflict of interest to declare.
